# Selective removal of some heavy metals from Lanthanide solution by graphene oxide functionalized with sodium citrate

**DOI:** 10.1038/s41598-022-17949-8

**Published:** 2022-08-12

**Authors:** E. M. Abu Elgoud, A. I. Abd-Elhamid, Sh. Sh. Emam, H. F. Aly

**Affiliations:** 1 Hot Laboratories Center, Egyptian Atomic Energy Authority, Cairo, 13759 Egypt; 2 Composites and Nanostructured Materials Department, Advanced Technology and New Materials Research Institute, City of Scientific Research and Technological Applications (SRTA-City), New Borg Al-Arab, Alexandria, 21934 Egypt

**Keywords:** Environmental sciences, Materials science

## Abstract

Lanthanides are widely used in several advanced technologies, and the presence of heavy metal ions as traces reduce their efficiencies. Hence, adsorption of some heavy metals from Lanthanides aqueous solution using previously prepared graphene oxide-citrate (GO-C) composite was reported. In this regard, the GO-C was applied to remove various heavy metal ions (Fe, Ni, Mn) through the batch technique. The GO-C after the adsorption process was characterized by various advanced techniques. The results obtained from the experimental investigations revealed that the GO-C showed a rapid equilibrium adsorption time (1.0 min) for all the studied heavy metal ions. Moreover, the adsorption isotherm data for Fe^3+^, Mn^2+^, and Ni^2+^ was fit by the Langmuir isotherm model with excellent adsorption capacity for Fe^3+^ (535.0 mg/g), Mn^2+^ (223.22 mg/g), and Ni^2+^ (174.65 mg/g). Furthermore, the GO-C can be reused over five times to enhance the removal efficiency. Interestingly, the GO-C adsorbent achieved removal performance reached 95.0% for the Fe^3+^ and ≥ 35.0% for Ni, Mn, Co, and Cu compared to < 1% for lanthanides metal ions.

## Introduction

Lanthanide’s elements have been used for several applications in advanced technologies, such as special ceramics and organic synthesis, in addition to their usages in some devices, such as batteries, sensors, energy-efficient lighting, and nuclear technologies. The presence of heavy metals in the lanthanides, even at minor concentrations, will shield its efficiency. Therefore, removing heavy metals, such as Fe^3^, Ni^2+^, and Mn^2+^ from lanthanide solution is very interesting. Moreover, toxic heavy metal contamination has become one of the most serious environmental problems. Where the heavy metal ions stir at extremely high speed in the watery circles, increasing their toxicity^1^. Iron and manganese often occur together in groundwater, but the concentration of manganese is usually much lower than the concentration of iron^2^. Iron may be present due to the utilization of the iron coagulants for the corrosion of steel and cast-iron pipes during groundwater extraction and distribution^3^. Nickel is a highly toxic heavy metal emitted to the environment from metallurgical, pesticides, electrolysis, electroplating, nuclear power plant, and mining operations^4^. Exposure to nickel may cause lung, nose, and bones cancer^5^. In addition, ^63^Ni is an important isotope created from the neutron activation of the reactor materials and is also more widely applied in nuclear research and medical applications^6^. Many techniques, such as chemical oxidation, ion exchange, co-precipitation, membrane filtration, electrochemical treatment, extraction, reverse osmosis, and adsorption, have been investigated to remove heavy and precious metals from wastewater. Among these methods, adsorption has been considered an engaging method and greatly used in industries due to its low cost and high efficiency, simplicity of design and operation, fast response, insensitivity to toxic pollutants, and smaller amounts of harmful substances^7^. Compared to many adsorbents, such as activated carbon^8^, neem leaf^9^, ferromagnetic carbon^10^, red mud^11^, alginate^12^, conducting polymers^13^, carbon nanotubes^14^, chitosan^15,16^, and ethyl cellulose^17,18^, graphene oxide (GO) is considered as the maximum favorable absorbent to adsorb different heavy metal ions^19,20^. This adsorption ability is due to its great specific surface area, hydrophilicity, high negative charge density and easily produced from the abundant natural graphite on a large-scale using chemical oxidation and peeling method^21,22^. Various materials have been utilized to modify graphene oxide to improve its adsorption selectivity and capacity, such as ethylene di-amine tetra acetic acid^23^, sulfanilic acid^24^, triethylenetetramine^25^, and polypyrrole^26^, etc.

Adsorption processes of graphene oxide and its composites have been reviewed^27–29^. Removal of Mn(II) by sodium alginate/graphene oxide composite double-network hydrogel beads from aqueous solutions has been studied by Yang et al.^30^. Their results showed that the graphene oxide/sodium alginate exhibited an excellent adsorption capacity of 56.49 mg/g. Adsorption of some heavy metal ions from aqueous single metal solutions on graphene oxide membranes has also been investigated by Tanet et al.^31^. Their results indicated that the maximum adsorption capacities of the GO membranes for Cu^2+^, Cd^2+^, and Ni^2+^ were approximately 72.6, 83.8, and 62.3 mg/g, respectively. Najaf et al. explored the adsorption of nickel ions from the aqueous phase using graphene oxide and glycine functionalized graphene oxide. They reported that the adsorption capacities of Ni^2+^ were estimated to be 38.61 and 36.63 on graphene oxide and glycine functionalized graphene oxide (GO-G), respectively^32^. Furthermore, the removal of nickel ions by graphene-MnO_2_ composite has been investigated by Renet al.^33^. Their findings showed that the saturation adsorption capacity of Ni(II) was 46.6 mg/g at room temperature. Moreover, Change et al.^34^ studied the adsorption of Fe^2+^ by graphene sheets. The obtained results revealed that the Fe^2+^ absorption capacity was 299.3 mg/g. Additionally, adsorption of some heavy metal ions, such as Cu^2+^, Zn^2+^, Fe^3+^, Pb^2+^, and Cr^3+^ has been investigated using poly(amidoamine) modified graphene oxide by Yuan et al.^35^. They reported that the maximum sorption capacity was 0.5312, 0.0798, 0.2024, 0.0513, and 0.1368 mmol/g for Fe^3+^, Cr^3+^, Zn^2+^ Pb^2+^, and Cu^2+^, respectively. Lei et al.^36^ evaluated the adsorption capacities of several heavy metals, such as Zn^2+^, Fe^3+^, Pb^2+^, and Cd^2+^ on foam-infused GO. Their results implied that the optimum adsorption capacities were 252.5, 381.3, 587.6, and 326 mg/g for Cd^2+^, Pb^2+^, Fe^3+^, and Zn^2+^, respectively.

Citric acid provides seven O-donor centers, which can be geometrically arranged around the metal ions for an efficient chelation process. Moreover, it is considered a favorable agent in the potentially beneficial compounds engineering, such as monomeric, binuclear, and polymeric complexes with both d- and f-electron metal ions. In our previous work^37^, we noted the superior properties of the as-prepared graphene oxide-citrate (GO-C) composite in removing cationic (organic and inorganic) species. Therefore, we suggested to extend our work to use the previously used composite (GO-C) in the selective removal of some heavy metal ions from the lanthanide aqueous solution. Therefore, the sorption behavior of (GO-C) towards some highly troublesome metal ions, such as Fe^3+^, Mn^2+^, and Ni^2+^ from aqueous solution using the batch technique was examined. Finally, the adsorption selectivity of GO-C towards Fe^3+^, Ni, Mn, Co, and Cu from lanthanides metal ions was tested. Moreover, the GO-C-M complex was characterized by advanced techniques after the adsorption process.

## Experimental

### Materials and instrumentation

The chemicals were of analytical grade and utilized without further purification. H_2_SO_4_ (95–97%, Riedel deHaen), H_2_O_2_ (36%, Pharaohs Trading and Import), HCl (30%, El Salam for Chemical Industries), KMnO_4_ (99%, Long live), and graphite (200 mesh, 99.99%, Alpha Aesar). Iron chloride (FeCl_3_) (Sigma-Aldrich), MnSO_4_·H_2_O (99%, Sigma-Aldrich), NiSO_4_·6H_2_O (99%, Sigma-Aldrich), tri-sodium citrate (Sigma-Aldrich), tetraethylorthosilicate (TEOS) (99%, Across), ethanol absolute (Sigma-Aldrich).

Analytical balance (CP 2245, Sartorius, USA.), Hot plate stirrer (IKA, C-MAG HS7, IKA®-Werke GmbH & Co. KG, Germany), pH meter (3510, Genway), Hot plate stirrer (SB 162, Stuart, UK.), and Centrifuge, (Mikro 220R, Hettich, UK.) were used.

### Preparation and characterization of GO-C composite

The composite was prepared according to our previous work^37^. The properties of citrated modified graphene oxide after adsorption were studied using SEM, EDX, FT-IR and Raman Spectroscopy. Surface morphology was identified using a JEOL SEM Model, JSM-6510A, Japan. The IR investigations were performed using an FTIR spectrometer, PerkinElmer, model 1600, USA. The elemental composition of Ni(II), Mn(II), and Fe(III) sorption on citrate-modified graphene oxide was detected by an Oxford energy-dispersive X-ray (EDX) spectrometer (Oxford Link ISIS, Japan). A Shimadzu UV–Visible double beam spectrophotometer (model UV-160A, Japan), was used for all spectrophotometric measurements.

### Batch sorption procedure

An iron (III) (1.0 g/L) stock solution was prepared by dissolving a known amount of iron chloride in minimum concentrated hydrochloric acid and evaporating it to near dryness, then formed to the mark with double-distilled water. Manganese and nickel solutions and standards (1.0 g/L) were prepared by dissolving a certain amount of manganese sulfate monohydrate and nickel sulfate hexahydrate in distilled water. The required concentrations of test solutions were prepared by appropriate dilution of the stock solutions.

The heavy metal ion concentrations of Ni^2+^ and Mn^2+^ were separately determined using the 4-(pyridyl-2-azo) resorcinol (PAR) method^38^. The concentration of iron was also determined using the thiocyanate method^38^. Batch sorption experiments were carried out by shaking 2.4 g (~ 0.2 mL) of the prepared citrated graphene oxide with 5.0 mL of known concentration of each metal ion aqueous solution in a thermostatic shaker bath at a constant temperature for a predetermined period. Metal ions adsorption was calculated as the difference between initial metal ions concentration in solution and its concentration after shaking time (t). The number of ions retained in the solid phase at equilibrium (q_e_) in (mg/g) was calculated using the following equation:

1 $${\text{q}}_{{\text{e}}} = \, \left( {{\text{C}}_{{\text{o}}} {-}{\text{ C}}_{{\text{e}}} } \right) \, \times {\text{ V}}/{\text{m,}}$$

where C_o_ and C_e_ are the initial and equilibrium concentrations in (mg/L) of ions solution, respectively, V is the volume of solution in (L), and m is the weight of the adsorbent in (g).

#### Kinetic study

See Supplementary Materials.

#### Adsorption isotherm

See Supplementary Materials.

#### Thermodynamic isotherm

See Supplementary Materials.

## Results and discussion

Preliminary investigations showed that citrate-modified graphene oxide (GO-C) can eliminate heavy metals from aqueous solution due to active functional groups (carboxylic group) of the citrate. Therefore, sorption investigations of the relevant metal ions were performed by the (GO-C) from an aqueous solution.

### Characterization of modified GO-C-M

Different techniques, such as SEM, FTIR, Raman, and EDX analysis, were used to characterize the citrate-modified graphene oxide-heavy metal ions (GO-C-M) complex to assess the adsorption process.

#### SEM analysis

The morphology of the GO-C composite was previously investigated by SEM and TEM techniques^37^. The SEM images presented that, the GO-C composite appears as a layered structure of GO loaded with the modifier. Further, the TEM images indicated that the GO-C is composed of the fully separated layered structure of GO decorated with dark spots of the modifier^37^.

**Figure 1:**
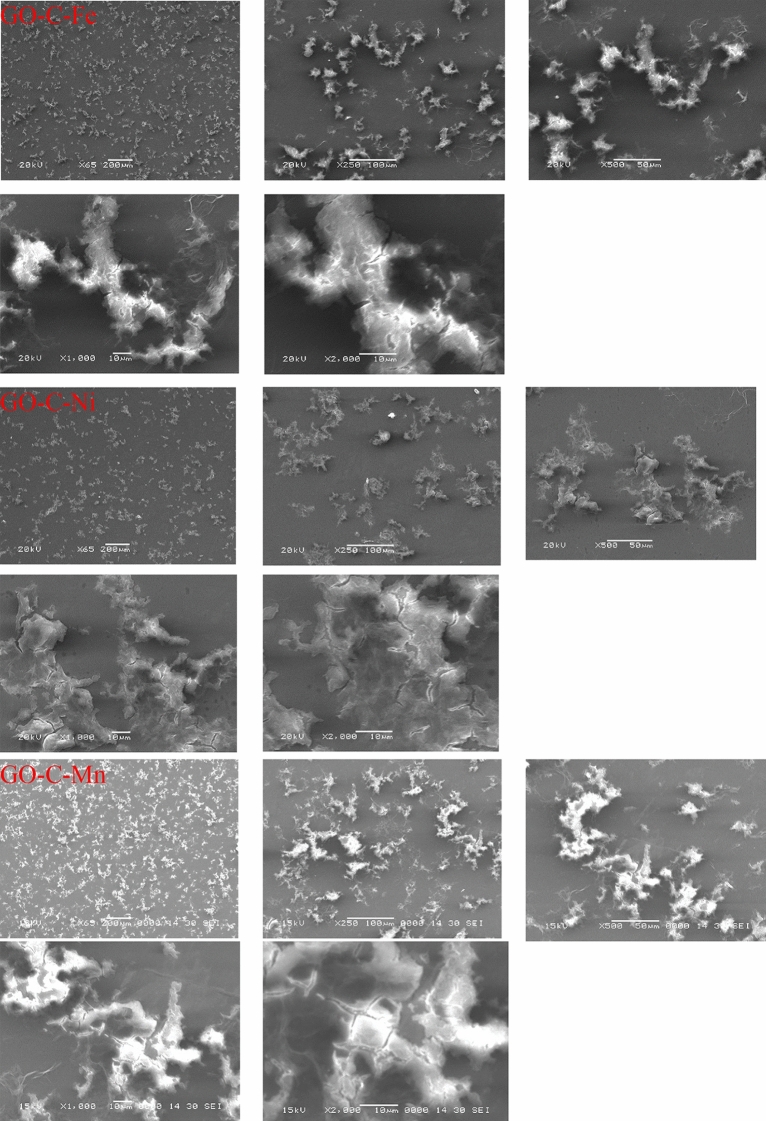
SEM images for GO-C-Fe complex, GO-C-Ni complex, and GO-C-Mn complex at different magnification.

The SEM images of the adsorbent-metal ion complexes (GO-C-M, M = Fe, Ni, or Mn) are presented in Fig. 1. The GO sheets were modified with sodium citrate, which has three full ionized carboxylate groups. These groups exhibited high interaction affinity with the metal ions. Therefore, introducing the GO-C in an aqueous solution of heavy metal ions tends to form a strong complex with this metal ion. This behavior changed the flat morphology of the GO-C to shrinkage structure of GO-C-M, as seen in Fig. 1.

### FT-IR analysis

**Figure 2:**
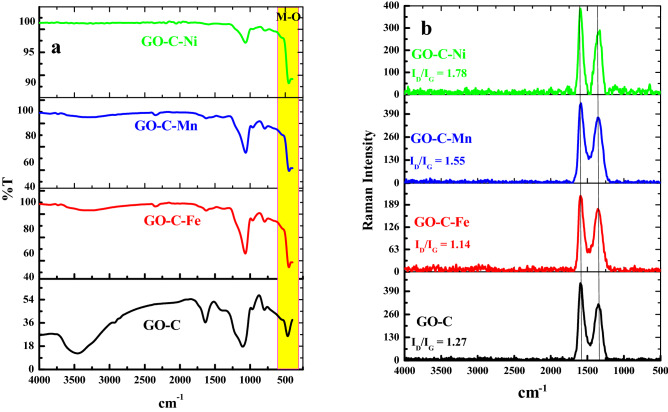
(**a**) FT-IR and (**b**) Raman spectra for GO-C composite, GO-C-Fe complex, GO-C-Mn complex, and GO-C-Ni complex.

The FTIR spectra of GO-C and GO-C-M (M = Fe^3+^, Ni^2+^, and Mn^2+^) are explored in Fig. 2a. The GO-C present bands typically as observed in the previous study^37^; at 3455 cm^−1^ (O–H stretching), 2931 cm^−1^ (C–H stretching of aliphatic CH_2_), 1637 cm^−1^ (O–H bending), 1381 cm^−1^ (COOH), 11,061 cm^−1^ (Si–O–Si asymmetric stretching vibrations), 797 cm^−1^ (symmetric stretching vibrations of Si–O–Si), as presented in Fig. 2a. The FITR spectra of the three complexes exhibit bands at 1625 cm^−1^ (OH), 1376–1387 cm^−1^ (O=C–O) 1069–1064 cm^−1^ (C–O) and (Si–O) and 793–787 cm^−1^ (M–O). Herein, the intensities of these peaks in GO-Cit-M highly reduced from the GO-C composite^39^ (Fig. 2a). Finally, a sharp intense band at 451 cm^−1^ corresponding to M–O bon stretching suggested the formation of a strong complex between the GO-C composite and M-ions.

#### Raman spectra

Raman spectroscopy is widely used to explore the structure change for GO to new functionalized graphene oxide. The Raman spectrum of GO^37^ shows two bands of D-band at 1352 cm^−1^ and the G-band at 1598 cm^−1^. It is well known that the G-band is related to the vibration of the sp^2^ carbon atoms in the graphitic 2D hexagonal lattice. On the other hand, the D-band reflects the disorder and local defects. This technique was used in terms of analysis of the location, intensities, and border of the D-band and G-band, as seen in Fig. 2b. The locations of the D and G-bands and the values of I_D_/I_G_ ratios and FWHMs are summarized in Table 1.

**Table 1:** The D and G-band positions, I_D_/I_G_ ratios, and FWHMs.

Sample	D-band peak		G-band peak		I_D_ /I_G_
	Raman shift (cm^−1^)	FWHM (cm^−1^)	Raman shift (cm^−1^)	FWHM (cm^−1^)	
GO-C	1351	112	1594	88	1.27
GO-C-Fe	1348	100	1598	88	1.136
GO-C-Ni	1352	137	1583	88	1.550
GO-C-Mn	1348	112	1594	63	1.780

In the case of adsorption of Fe^3+^ on the GO-C composite, the I_D_/I_G_ ratio of the GO-C-Fe was 1.136, which is less than the I_D_/I_G_ ratio for the GO-C composite (1.27). While in the case of Ni^2+^ and Mn^2+^, the I_D_/I_G_ ratio for GO-C-Ni and GO-C-Mn is 1.550 and 1.780, respectively. This result suggests that the type and the oxidation state of the metal ion affected the defect states (sp^2^/sp^3^ plane) of the GO-C composite.

#### EDX analysis

**Figure 3:**
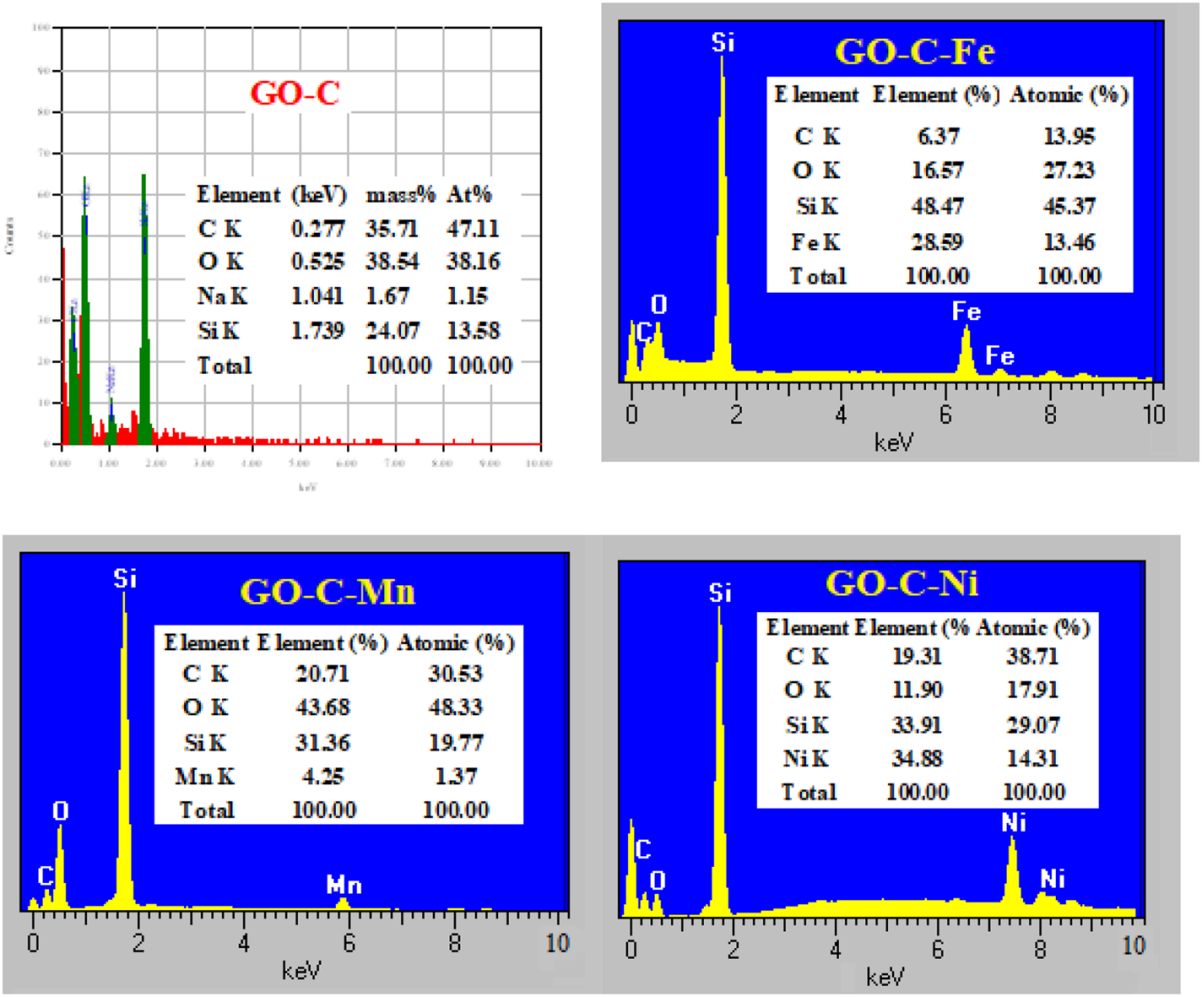
EDX analysis for GO-C composite, GO-C-Ni complex, GO-C-Fe complex, and GO-C-Mn complex.

The importance of EDS analysis highlights the elemental composition of the fabricated material. Graphene oxide is a carbonaceous material mainly composed of C and O-atoms. Herein, we modified the GO with tri-sodium citrate and used tetraethylorthosilicate (TEOS) as a binder. Therefore, the elemental analysis of GO-C shows the presence of Na and Si atoms in the resulting EDS analysis^37^, see Fig. 3. When GO-C was used in treating aqueous solutions of Fe^3+^, Ni^2+^, and Mn^2+^, the M ions were expected to bind with the composite at the carboxylate groups (–COO^−^Na^+^) to form (–COO^−^M^+^) and release the Na^+^. Hence, in the analysis of the GO-C-M, the M-ions will appear in the results instead of Na^+^, as presented in Fig. 3.

### Batch investigations

Preliminary batch investigations were carried out to assess the time required for the adsorption equilibrium, pH, V/m ratio for statistically acceptable adsorption values, as well as the effect of the initial metal ions concentrations and temperature.

#### Effect of contact time

**Figure 4:**
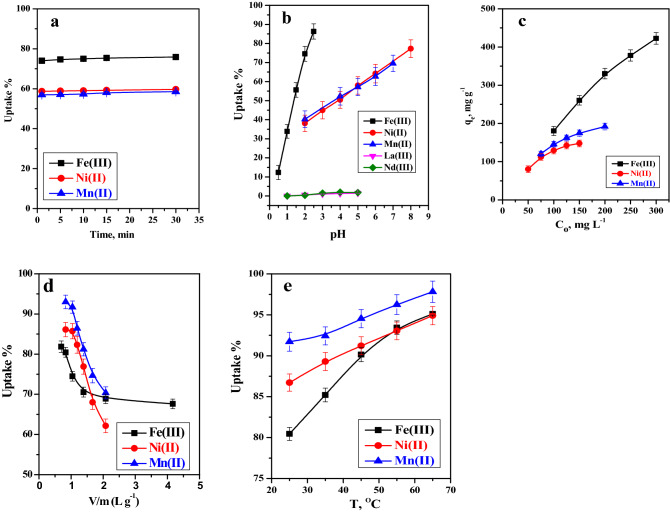
Effect of (**a**) contact time on uptake percent of Fe^3+^([Fe] = 100 mg/L, pH = 2), Ni^2+^([Ni] = 50 mg/L, pH = 5) and Mn^2+^([Mn] = 100 mg/L, pH = 5) from aqueous media, V/m = 2.1 L/g, T = 25 °C, (**b**) Effect of aqueous solution pH on uptake percent of 100 mg/L Fe^3+^, 50 mg/L Ni^2+^, 100 mg/L Mn^2+^, 100 mg/L La^3+^ and 100 mg/L Nd^3+^ from aqueous media, V/m = 2.1 L/g, T = 25 °C after 1.0 min, (**c**) Relation between the adsorbed amount, q_e_, of Fe (pH = 2.5), Mn (pH = 7.0) and Ni (pH = 8.0) and the initial concentrations of these ions, V/m = 2.1 L/g, T = 25 °C after 1.0 min, (**d**) effect of V/m ratio on uptake percent of Fe^3+^([Fe] = 300 mg/L, pH = 2.5), Ni^2+^([Ni] = 100 mg/L, pH = 8) and Mn^2+^([Mn] = 100 mg/L, pH = 7) from aqueous media, T = 25 °C after 1.0 min, (**e**) effect of temperature on uptake percent of Fe^3+^([Fe] = 300 mg/L, pH = 2.5, V/m = 0.83 L/g), Ni^2+^([Ni] = 100 mg/L, pH = 8, V/m = 1.04 L/g) and Mn^2+^([Mn] = 100 mg/L, pH = 7, V/m = 1.04 L/g) from aqueous media, after 1.0 min.

In practical application, the adsorption contact time is a very important factor. The influence of the contact time (1.0–30.0 min) on the uptake percent of Fe^3+^ (100.0 mg/L, pH = 2.0), Ni^2+^ (50.0 mg/L, pH = 5.0), and Mn^2+^ (100.0 mg/L, pH = 5.0) and V/m ratio was kept at 2.1 L/g for all metal ions using the GO-C was plotted in Fig. 4a. The adsorption behavior recorded a high removal efficiency in the early stages (1.0 min) and remained nearly constant with the further increase in the contact time. This fast adsorption may be related to the flat structure of the composite^37^, which makes a large number of the carboxylated functional (–COO^−^Na^+^) exposed to the adsorbed metal ions. Moreover, the carboxylated groups tend to form a complex with the M-ion.

#### Effect of aqueous solution pH

The initial pH of the solution is a significant parameter that stimulates the adsorption process. It is affected not only by the adsorbent surface charge but also by the degree of the adsorbate ionization. Here, the effect of pH aqueous solution on the uptake percent for the studied metal ions in the range (0.5–2.5 for Fe^3+^, 2.0–8.0 for Ni^2+^, 2.0–7.0 for Mn^2+^, 1.0–5.0 for La^3+^, and 1.0–5.0 for Nd^3+^) was investigated and plotted in Fig. 4b. As indicated, the uptake percent of the studied metal ions (Fe^3+^, Ni^2+^, and Mn^2+^) is linearly increased with the further increase in solution pH values. Accordingly, pH values 2.0 for Fe^3+^, 8.0 for Ni^2+^ and 7.0 for Mn^2+^ were chosen for further investigations. While the uptake percent for La^3+^ and Nd^3+^ did not exceed 2.0% at different pH values. Therefore, pH = 2.0 was chosen to optimize the purification of La(III) and Nd(III) from Fe(III), Ni(II), and Mn(II).

As previously proposed^37^, the used GO-C composite contains three sodium carboxylate groups in the solid phase. In an aqueous solution at low pH, the sodium ions will exchange with the H^+^ in the solution. This will form mono and diprotonated citrate on the surface of the composite. Based on the Medusa program (www.kemi.kth.se/medusa), the speciation diagram for citric acid as a function of pH is given in Fig. S1. From this figure (Fig. S1), the main citrate species is present as H_2_(Cit)^−^ and H(Cit)^2−^ at pH = 2.0. Consulting the citrate complexes of metal ions under-investigated, it is reported that Fe^3+^, Ni^2+^, and Mn^2+^ form stable complexes with monoprotonated citrate anions^40–42^. This explains the sorption of these metal cations on the prepared composite at a low hydrogen ion concentration. Further, at pH = 3.0 Fe^3+^ donates precipitate. As for the lanthanides element the main complexes formed are with hydrated citrate anions^43^ and they have limited complexing ability to mono and deprotonated complexes.

#### Effect of initial metal ions concentrations

The relation between the adsorbed amount (q_e_, mg/g) and the initial metal ion concentration (C_o_, mg/L) in the rang (100.0–300.0 for Fe^3+^, 50.0–150.0 for Ni^2+^, and 75.0–200.0 for Mn^2+^) of the tested metal ions using GO-C composite is given in Fig. 4c. It is observed that the amount of the adsorbed metal ions (Fe^3+^, Ni^2+^, and Mn^2+^) increases with the increase of the initial concentration of the metal ions in the tested range. This observation can be indicated by increasing the initial metal ion concentration, which leads to an increase in the concentration gradient, which is performed as a driving force to reduce the resistance to mass transfer of the metal ion from the bulk of solution to the adsorbent surface. Then, the affinity of the binding sites for interaction with the metal ions increases, and thus, the adsorption capacity is enhanced.

#### Effect of V/m ratio

In order to evaluate the optimum GO-C weight, which donated the highly acceptable adsorption values, the induced V/m ratio (L/g) in the range (4.2–0.7 for Fe^3+^, and 2.1–0.83 for both Ni^2+^ and Mn^2+^) on the uptake percent of the studied metal ions from aqueous solution was investigated (Fig. 4d). The uptake percent was increased as the V/m ratio decreased for the three metal ions. The optimum V/m ratio was chosen at 0.83 L/g for Fe^3+^ and 1.04 L/g for both Ni^2+^ and Mn^2+^.

#### Effect of temperature

The effect of temperature (25–65 °C) on the uptake percent of (Fe^3+^, Ni^2+^, and Mn^2+^) ions from their aqueous solutions using GO-C composite was graphed in Fig. 4e. It is obvious that the uptake percent of the three metal ions increases with increasing the media temperature. This result demonstrated that the adsorption of the studied metal ions using the (GO-C) is an endothermic process. Moreover, the increase in the aqueous solution temperature enhanced the metal ion movement from the bulk of the solution to be closer to the chelating sites, which facile the adsorption process.

### Adsorption kinetic model, sorption isotherm model, and thermodynamics

**Figure 5:**
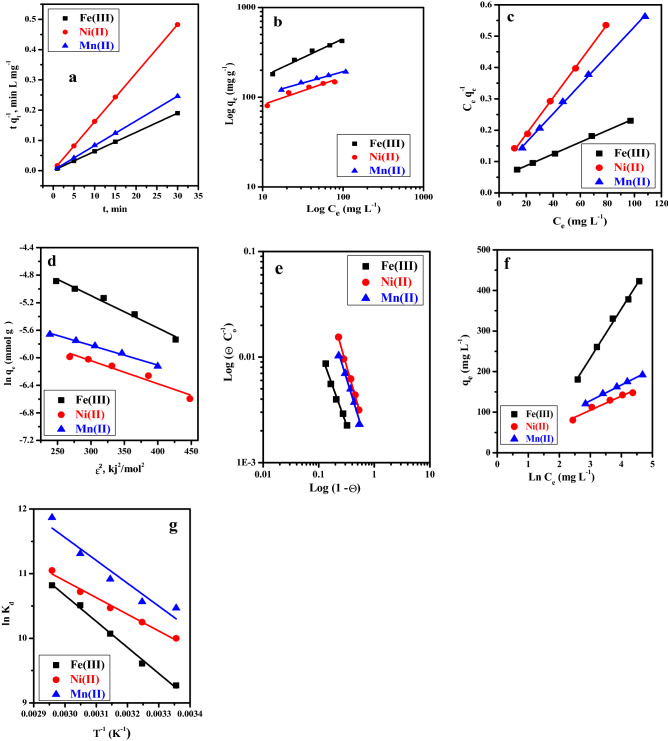
(**a**) Pseudo second-order plots, for the adsorption of Fe^3+^([Fe] = 100 mg/L, pH = 2), Ni^2+^([Ni] = 50 mg/L, pH = 5) and Mn^2+^([Mn] = 100 mg/L, pH = 5) from aqueous media, V/m = 2.1 L/g, T = 25 °C using the modified (GO-C), (**b**) linear Freundlich isotherm plots, (**c**) linear Langmuir isotherm plots, (**d**) linear D-R isotherm plots. (**e**) Linear Flory–Huggins isotherm plots, (**f**) linear Temkin isotherm plots for the sorption of Fe^3+^(pH = 2.5), Ni^2+^(pH = 8) and Mn^2+^(pH = 7) from aqueous media, V/m = 2.1 L/g, T = 25 °C using the modified (GO-C) after 1.0 min and (**g**) Thermodynamic parameters for the adsorption of Fe^3+^([Fe] = 300 mg/L, pH = 2.5, V/m = 0.83 L/g), Ni^2+^([Ni] = 100 mg/L, pH = 8, V/m = 1.04 L/g) and Mn^2+^([Mn] = 100 mg/L, pH = 7, V/m = 1.04 L/g) from aqueous media, after 1.0 min.

The adsorption kinetics were investigated to assess the rate-controlling step, mass transfer, and chemical reaction process. As presented in the effect of mixing time section, the adsorption equilibrium reached high rapidly (1.0 min). Therefore, the adsorption kinetics were investigated employing the pseudo-second-order (see Supplementary Materials). The linear relation between the t and t/q_t_ was plotted in Fig. 5a, and different parameters were calculated and listed in Table 2. It was observed that the correlation coefficient is R^2^ ≥ 0.995. Moreover, the calculated adsorption capacity was closer to the experimental adsorption capacities values. These findings suggested that the adsorption kinetics are excellently fitted with pseudo-second-order, which indicates that chemical adsorption is more predominant. This result can illustrate that the mechanism of Fe^3+^, Ni^2+^, and Mn^2+^ onto GO-C is controlled by the exchange mechanism.

**Table 2:** Calculated parameters of the linear pseudo-second-order kinetic models for Fe^3+^, Ni^2+^, and Mn^2+^ sorbed onto the modified (GO-C).

Metal ion	Linear pseudo-second order			
	q_e exp_ (mg/g)	q_e cal_ (mg/g)	k_2_ (g/mg/min)	R^2^
Fe (III)	158.063	158.23	75.25 × 10^–3^	0.999
Ni (II)	62.18	62.27	203.07 × 10^–3^	0.999
Mn (II)	121.96	122.25	62.53 × 10^–3^	0.999

Adsorption isotherm is important to design the adsorption systems. Moreover, it explains the relationship between the amount of adsorbate uptake from the aqueous phase using a unit mass of the adsorbent at a constant temperature. Equilibrium isotherm modeling was performed using Langmuir, Freundlich, Dubinin–Radushkevich, Temkin, and Flory–Huggins isotherms (see Supplementary Materials). Furthermore, the linear isotherm modeling plots are shown in Fig. 5b–f, respectively. The correlation coefficient and adsorption isotherm parameters of different models were evaluated and summarized in Table 3.

**Table 3:** Calculated parameters of the linear Freundlich, Langmuir, D-R, Flory–Huggins and Temkin isotherm models for Fe^3+^, Ni^2+^, and Mn^2+^ sorbed onto the modified (GO-C).

Isotherm models	Metal ions	q_oexp_. mg/g	Isotherm models parameters			
			K_f_ (mg/g)	1/n		R^2^
Freundlich	Fe(III)	535.0	64.13	2.37		0.963
	Ni (II)	174.65	40.56	3.24		0.917
	Mn(II)	223.22	60.58	3.98		0.978

			Q_o_ (mg/g)	b (mL/mg)	R_L_	R^2^
Langmuir	Fe(III)		531.91	0.0385	0.206	0.999
	Ni (II)		171.23	0.0833	0.107	0.999
	Mn(II)		216.45	0.0679	0.128	0.999

			*q*_*m,*_ mmol/g	*β,* mol^2^/kJ^2^	*E*, kJ/mol	R^2^
D-R isotherm	Fe(III)		0.0251	− 0.0047	10.31	0.978
	Ni (II)		0.0065	− 0.00334	12.235	0.938
	Mn(II)		0.0071	− 0.00287	13.199	0.989

			n_FH_	K_FH_	ΔG°, kJ/mole	R^2^
Flory–Huggins	Fe(III)		− 1.46669	0.00042	19.254	0.983
	Ni (II)		− 1.83308	0.001	17.109	0.993
	Mn(II)		− 1.71077	0.00087	17.449	0.981

			q_e_,_cal_, mg/g	b_T_	K_T_, L/g	R^2^
Temkin	Fe(III)		424.57	20.454	0.343	0.995
	Ni (II)		152.45	71.459	1.028	0.961
	Mn(II)		193.36	64.088	1.378	0.996

We noted that the values of R^2^ for all the studied metal ions (Fe^3+^, Ni^2+^, and Mn^2+^) related to the Langmuir model were (0.999) closer to the unit. Moreover, the maximum adsorption capacities, mg/g, were 531.91 (Fe^3+^), 171.23 (Ni^2+^), and 223.22 (Mn^2+^). Moreover, the sorption Langmuir energy (b) values for the metals studied were greater than zero, explaining that Langmuir is the appropriate model. The R_L_ values were < 1.0, and > 0 indicates high favorable sorption of Fe^3+^, Ni^2+^, and Mn^2+^ on GO-C for all studied concentrations. Moreover, according to the error function data in Table S1, it is clear that Langmuir is the best model to describe the adsorption data. The Langmuir isotherm assumes that the solid surface has a finite number of identical sites that are energetically uniform. According to the Langmuir model, there is no interaction between adsorbed species, which means that the adsorbed amount did not influence the adsorption rate. A monolayer was formed when the equilibrium was attained.

To further optimize the thermodynamic parameters (see Supplementary Materials) of the adsorption process, Gibbs free energy (ΔG°), Enthalpy (ΔH°), and Entropy (ΔS°) were detected related to Fig. 5g, and the measured parameters are listed in Table 4. The increase in negative values of the ΔG° with a further increase in the temperature reveals that the metal ions interact spontaneously with the GO-Cit surface. On the other hand, the values of ΔH° and ΔS° are tabulated in Table 4. This table shows that the positive values of ΔH° refer to the endothermic type of the sorption process, while the positive values of (ΔS°) show an increase in the randomness of the system. Moreover, the affinity of the GO-C towards the metal ion increase with temperature rises.

**Table 4:** Thermodynamic parameters for Fe^3+^, Ni^2+^ and Mn^2+^ removal from aqueous solution using GO-C composite.

Metal ions	T(K)	ΔG° (k J/mole)	ΔH° (k J/mole)	ΔS° (J/mole/K )
Fe (III)	298	− 22.97	33.49	189.13
	308	− 24.61		
	318	− 26.62		
	328	− 28.66		
	338	− 30.41		
Ni(II)	298	− 24.78	21.46	154.88
	308	− 26.25		
	318	− 27.68		
	328	− 29.23		
	338	− 31.05		
Mn (II)	298	− 25.93	29.41	184.32
	308	− 27.06		
	318	− 28.86		
	328	− 30.84		
	338	− 33.35		

### Regeneration and reusability

**Figure 6:**
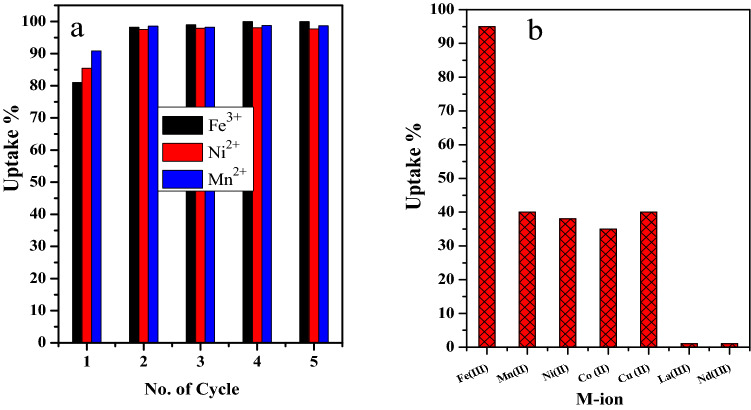
(**a**) The effect of the number of the re-use cycles of the GO-C on the uptake percentage of Fe^3+^ (t = 1.0 min, [Fe] = 300 mg/L, Dose = 6 mg, V = 5.0 mL, pH = 2.5,T = 25 °C), Ni^2+^([Ni] = 100 mg/L, Dose = 4.8 mg, V = 5.0 mL, pH = 8, T = 25 °C) and Mn^2+^([Mn] = 100 mg /L, Dose = 4.8 mg, V = 5.0 mL, pH = 7, T = 25 °C) from aqueous media, and (**b**) removal of some heavy metal ions mixture from lanthanides solution. ([Fe^3+^] = 300.0 mg/L; [Mn^2+^] = 100.0 mg/L; [Ni^2+^] = 100.0 mg/L; [Co^2+^] = 100.0 mg/L; [Cu^2+^] = 100.0 mg/L, [La^3+^] = 100.0 mg/L; [Nd^3+^] = 100.0 mg/L V = 10.0 mL, m = 0.024 g, pH = 2, Shaking time = 1.0 min, T = 25 °C).

The ability to release the adsorbed metal ion from the binding site on the adsorbent is a significant factor in evaluating the economic efficiency and applicability of the adsorbent used. Thus, the regeneration of the GO-C composite was studied. Herein, the adsorbent firstly adsorbed the M-ion. Secondly, 10.0% of HCl was selected to liberate the M-ion from the adsorbent binding site and washed with distilled water. Finally, the GO-C composites were activated with 10.0% NaOH and washed with distilled water. The regenerated GO-C was reused to adsorb the metal ion again, as illustrated in Fig. 6a. It was obviously noted that the regenerated GO-C composite shows a little higher removal efficiency than that of pristine composite, which increases the composite evaluability and applicability.

### Selective adsorption of different heavy metal ions from lanthanide aqueous solution

It was highly interesting to study the removal efficiency of a mixture of metal ions like Fe^3+^, Ni^2+^, Mn^2+^, Co^2+^, and Cu^2+^ from lanthanides (La (III) and Nd (III) solution) using the regenerated GO-C adsorbent at pH = 2, as present in Fig. 6b. The experimental results showed that the GO-C composite showed a high adsorption affinity towards the studied heavy metal ions in the presence of the lanthanides ions. Whereas the GO-C adsorbent achieved removal performance reached 95.0% for the Fe^3+^ and ≥ 35.0% for Ni, Mn, Co, and Cu compared to < 1% for lanthanide metal ions. This result indicated a high adsorption selectivity of some heavy metals from highly acidic solutions in the presence of lanthanide metal ions. Furthermore, the adsorbent owns the property to adsorb Fe^3+^ from metal ion mixture at low pH = 2.

### Comparison between the studied heavy metal ions onto other sorbents

The sorption capacity of the citrate-modified graphene oxide was compared with other sorbents reported in the literature (Table 5). The data showed that the citrate-modified graphene oxide showed a significantly higher adsorption capacity for the studied metal ions. Therefore, it can be considered a highly effective material to adsorb these metals from an aqueous solution.

**Table 5:** Comparison of capacity values for the studied heavy metal ions adsorbed by various sorbents.

Metal ion	Adsorbent	Q_o_, mg/g	Ref.
Ni(II)	Citrate-modified graphene oxide	174.65	This work
	Activated carbon prepared from coirpith by chemical activation (Carbonised coirpith)	62.5	^44^
	peat	61.27	^45^
	Amberlite IR-120 resin	48.07	^46^
	Clinoptilolite	13.03	^47^
	Wheat straw	41.84	^48^
	Barley straw	35.80	^49^
	Tea factory waste	18.42	^50^
Fe(III)	Citrate modified graphene oxide	535.0	This work
	Prepared activated carbon	6.54	^51^
	Coir fibres	2.84	^52^
	Modified coir fibres	7.49	^52^
	Activated carbon from coconut shells	81.89	^53^
	Chitosan/polyethylene glycolblend membrane	90.9	^54^
	Chitosan	57.5	^55^
	Rice husk ash	6.21	^56^
	Thiosalcylic acid (TSA)	275.78	^57^
Mn(II)	Citrate modified graphene oxide	223.22	This work
	Rice husk ash	3.02	^56^
	Activated carbon fromcoconut shells	75.65	^53^
	Chitosan/polyethylene glycolblend membrane	21.7	^54^
	Crab shell particles	69.9	^58^
	Tannic acid immobilized activated carbon	1.13	^59^
	Manganese oxide coatedzeolite	1.1	^60^
	Prepared activated carbon	4.72	^51^

## Conclusion

Citrate-modified graphene oxide (GO-C) was investigated to remove some heavy metals from lanthanides solution and characterized before and after adsorption using SEM, FTIR, Raman, and EDX. The modified GO-C showed rapid kinetics and an excellent adsorption capacity for Mn^2+^ (223.22 mg/g), Fe^3+^ (535.0 mg/g), and Ni^2+^ (174.65 mg/g). The adsorption process using the modified (GO-C) is an endothermic and spontaneous reaction. Moreover, the GO-C can be reused over five times to enhance the efficiency of the removal process. In addition, the GO-C composite can achieve removal efficiency of 95.0% for the Fe^3+^ and ≥ 35.0% for Ni, Mn, Co, and Cu compared to < 1% for lanthanides metal ions. Within this approach, the separation of pure lanthanides for technological application will add economic value to the water treatment process.

## Supplementary Information

Supplementary Information.

## Data Availability

All data generated or analyzed during this study are included in this published article and its supplementary information files.

## References

[1] Blazquez A, Martin MA, Lara MC, Marti R, Campos Y, Cabello A, Cabello A, Garesse R, Bautista J, Andreu AL, Arenas J (2005). Removal of cadmium ions with olive stones: The effect of some parameters. Process Biochem..

[2] El Araby R, Hawash S, El Diwani G (2009). Treatment of iron and manganese in simulated groundwater via ozone technology. Desalination.

[3] García-Mendieta A, Solache-Ríos M, Olguín MT (2009). Evaluation of the sorption properties of a Mexican clinoptilolite-rich tuff for iron, manganese and iron–manganese systems. Microporous Mesoporous Mater..

[4] Zhang H, Chen L, Zhang LP, Yu XJ (2010). Impact of environmental conditions on the adsorption behavior of radionuclide Ni(II) onto hematite. J. Radioanal. Nucl. Chem..

[5] Sobhanardakani S, Zandipakb R, Javad Mohammadi M (2016). Removal of Ni(II) and Zn(II) from aqueous solutions using chitosan. Arch. Hygiene Sci..

[6] Chen L, Yu S, Huang L, Wang G (2012). Impact of environmental conditions on the removal of Ni(II) from aqueous solution to bentonite/iron oxide magnetic composites. J. Radioanal. Nucl. Chem..

[7] Yang Y, Wu WQ, Zhou HH (2014). Adsorption behavior of cross-linked chitosan modified by graphene oxide for Cu(II) removal. J. Cent South Univ..

[8] Krishnan KA, Anirudhan TS (2002). Uptake of heavy metals in batch systems by sulfurized steam activated carbon prepared from sugarcane bagasse Pith. Ind. Eng. Chem. Res..

[9] Bhattacharyya KG, Sharma A (2005). Kinetics and thermodynamics of methylene blue adsorption on neem (*Azadirachta indica*) leaf powder. Dyes Pigments.

[10] Zhang D, Wei S, Kaila C (2010). Carbon-stabilized iron nanoparticles for environmental remediation. Nanoscale.

[11] Gupta V, Suhas IA, Saini V (2004). Removal of Rhodamine B, Fast green, and methylene blue from wastewater using red mud, an aluminum industry waste. Ind. Eng. Chem. Res..

[12] Abu Al-Rub FA, El-Naas MH, Benyahia F, Ashour I (2004). Biosorption of nickel on blank alginate beads, free and immobilized algal cells. Process Biochem..

[13] Mahanta D, Madras G, Radhakrishnan S, Patil S (2009). Adsorption and desorption kinetics of anionic dyes on doped polyaniline. J. Phys. Chem. B.

[14] Madrakian T, Afkhami A, Ahmadi M, Bagheri H (2011). Removal of some cationic dyes from aqueous solutions using magnetic-modified multi-walled carbon nanotubes. J. Hazard. Mater..

[15] Galhoum AA, Atia AA, Mahfouz MG (2015). Dy(III) recovery from dilute solutions using magnetic-chitosan nano-based particles grafted with amino acids. J. Mater. Sci..

[16] Liu B, Wang D, Xu Y, Huang G (2011). Adsorption properties of Cd(II)-imprinted chitosan resin. J. Mater. Sci..

[17] Qiu B, Guo J, Zhang X (2014). Polyethylenimine facilitated ethyl cellulose for hexavalent chromium removal with a wide pH range. ACS Appl. Mater. Interfaces.

[18] Qiu B, Xu C, Sun D (2014). Polyaniline coated ethyl cellulose with improved hexavalent chromium removal. ACS Sustain. Chem. Eng..

[19] Wang H, Yuan X, Wu Y, Huang H, Zeng G, Liu Y, Wang X, Lin N, Qi Y (2013). Adsorption characteristics and behaviors of graphene oxide for Zn(II) removal from aqueous solution. Appl. Surf. Sci..

[20] Zhao G, Ren X, Gao X, Tan X, Li J, Chen C, Huang Y, Wang X (2011). Removal of Pb(ii) ions from aqueous solutions on few-layered graphene oxide nanosheets. Dalton Trans..

[21] Gu D, Fein JB (2015). Adsorption of metals onto graphene oxide: Surface complexation modeling and linear free energy relationships. Colloids Surf. A Physicochem. Eng. Asp..

[22] Yang X, Chen C, Li J, Zhao G, Ren X, Wang X (2012). Graphene oxide-iron oxide and reduced graphene oxide-iron oxide hybrid materials for the removal of organic and inorganic pollutants. RSC Adv..

[23] Cui L, Wang Y, Gao L, Hu L, Yan L, Wei Q, Du B (2015). EDTA functionalized magnetic graphene oxide for removal of Pb(II), Hg(II) and Cu(II) in water treatment: Adsorption mechanism and separation property. Chem. Eng. J..

[24] Hu X-J, Liu Y-G, Wang H, Chen A-W, Zeng G-M, Liu S-M, Guo Y-M, Hu X, Li T-T, Wang Y-Q, Zhou L, Liu S-H (2013). Removal of Cu(II) ions from aqueous solution using sulfonated magnetic graphene oxide composite. Sep. Purif. Technol..

[25] Chen JH, Xing HT, Sun X, Su ZB, Huang YH, Weng W, Hu SR, Guo HX, Wu WB, He YS (2015). Highly effective removal of Cu(II) by triethylenetetramine-magnetic reduced graphene oxide composite. Appl. Surf. Sci..

[26] Chandra V, Kim KS (2011). Highly selective adsorption of Hg2+ by a polypyrrole-reduced graphene oxide composite. Chem. Commun..

[27] Chen D, Feng H, Li J (2012). Graphene oxide: Preparation, functionalization, and electrochemical applications. Chem. Rev..

[28] Peng W, Li H, Liu Y, Song S (2017). A review on heavy metal ions adsorption from water by graphene oxide and its composites. J. Mol. Liq..

[29] Ali I, Mbianda XY, Burakov A, Galunin E, Burakova I, Mkrtchyan E, Grachev V (2019). Graphene based adsorbents for remediation of noxious pollutants from wastewater. Environ. Int..

[30] Yang X, Zhou T, Ren B, Hursthouse A, Zhang Y (2018). Removal of Mn (II) by sodium alginate/graphene oxide composite double-network hydrogel beads from aqueous solutions. Sci. Rep..

[31] Tan P, Sun J, Hu Y, Fang Z, Bi Q, Chen Y, Cheng J (2015). Adsorption of Cu(2+), Cd(2+) and Ni(2+) from aqueous single metal solutions on graphene oxide membranes. J. Hazard. Mater..

[32] Najafi F, Moradi O, Rajabi M, Asif M, Tyagi I, Agarwal S, Gupta VK (2015). Thermodynamics of the adsorption of nickel ions from aqueous phase using graphene oxide and glycine functionalized graphene oxide. J. Mol. Liq..

[33] Ren Y, Yan N, Wen Q, Fan Z, Wei T, Zhang M, Ma J (2011). Graphene/δ-MnO2 composite as adsorbent for the removal of nickel ions from wastewater. Chem. Eng. J..

[34] Chang CF, Truong QD, Chen JR (2013). Graphene sheets synthesized by ionic-liquid assisted electrolysis for application in water purification. Appl. Surf. Sci..

[35] Yuan Y, Zhang G, Li Y, Zhang G, Zhang F, Fan X (2013). Poly(amidoamine) modified graphene oxide as an efficient adsorbent for heavy metal ions. Polym. Chem..

[36] Lei Y, Luo Y, Zhang L (2014). Synthesis of three-dimensional graphene oxide foam for the removal of heavy metal ions. Chem. Phys. Lett..

[37] Abd-Elhamid AI, Abu Elgoud EM, Emam SS, Aly HF (2022). Superior adsorption performance of citrate modified graphene oxide as nano material for removal organic and inorganic pollutants from aqueous solution. Sci. Rep..

[38] Marczenko Z (1986). Spectrophotometric Determination of Elements.

[39] Abd-Elhamid AI, Aly HF, Soliman HAM, El-Shanshory AA (2018). Graphene oxide: Follow the oxidation mechanism and its application in water treatment. J. Mol. Liq..

[40] Vukosav P, Mlakar M, Tomišić V (2012). Revision of iron (III)–citrate speciation in aqueous solution. Voltammetric and spectrophotometric studies. Anal. Chim. Acta.

[41] Silva AM, Kong X, Parkin MC, Cammack R, Hider RC (2009). Iron (III) citrate speciation in aqueous solution. Dalton Trans..

[42] Wyrzykowski D, Chmurzyński L (2010). Thermodynamics of citrate complexation with Mn2+, Co2+, Ni2+ and Zn2+ ions. J. Therm. Anal. Calorim..

[43] Abu Elgoud EM, Ismail ZH, El-Nadi YA, Aly HF (2020). Separation of cerium (IV) and yttrium (III) from citrate medium by solvent extraction using D2EHPA in kerosene. Chem. Pap..

[44] Kadirvelu K, Thamaraiselvi K, Namasivayam C (2001). Adsorption of nickel(II) from aqueous solution onto activated carbon prepared from coirpith. Sep. Purif. Technol..

[45] Bartczak P (2018). Removal of nickel(II) and lead(II) ions from aqueous solution using peat as a low-cost adsorbent: A kinetic and equilibrium study. Arab. J. Chem..

[46] Demirbas A, Pehlivan E, Gode F, Altun T, Arslan G (2005). Adsorption of Cu(II), Zn(II), Ni(II), Pb(II), and Cd(II) from aqueous solution on Amberlite IR-120 synthetic resin. J. Colloid Interface Sci..

[47] Chaouch N, Ouahrani MR, Chaouch S, Gherraf N (2013). Adsorption of cadmium (II) from aqueous solutions by activated carbon produced from Algerian dates stones of *Phoenix dactylifera* by H3PO4 activation. Desalin. Water Treat..

[48] Dhir B, Kumar R (2010). Adsorption of heavy metals by Salvinia biomass and agricultural residues. Int. J. Environ. Res..

[49] Thevannan A, Mungroo R, Niu CH (2010). Biosorption of nickel with barley straw. Bioresour. Technol..

[50] Malkoc E, Nahoglu Y (2005). Investigations of Ni(II) removal from aqueous solutions using tea factory waste. J. Hazard. Mater..

[51] El-Sherif IY, Fathy NA, Hanna AA (2013). Removal of Mn (II) and Fe (II) ions from aqueous solution using precipitation and adsorption methods. J. Appl. Sci. Res..

[52] Shukla SR, Pai RS, Shendarkar AD (2006). Adsorption of Ni(II), Zn(II) and Fe(II) on modified coir fibres. Sep. Purif. Technol..

[53] Moreno-Pirajan JC, Garcia-Cuello VS, Giraldo L (2011). The removal and kinetic study of Mn, Fe, Ni and Cu ions from wastewater onto activated carbon from coconut shells. Adsorption.

[54] Reiad NA, Salam OEA, Abadir EF, Harraz FA (2012). Adsorptive removal of iron and manganese ions from aqueous solutions with microporous chitosan/polyethylene glycol blend membrane. J. Environ. Sci..

[55] Ngah WS, AbGhani S, Kamari A (2005). Adsorption behaviour of Fe(II) and Fe(III) ions in aqueous solution on chitosan and cross-linked chitosan beads. Bioresour. Technol..

[56] Zhang Y, Zhao J, Jiang Z, Shan D, Lu Y (2014). Biosorption of Fe(II) and Mn(II) ions from aqueous solution by rice husk ash. BioMed Res. Int..

[57] Abd-Elhamid AI, Aly HF (2018). Removal of Fe (III) from aqueous solution using thiosalcylic acid as an efficient and novel adsorbent. Egypt. J. Chem..

[58] Vijayaraghavan K, Winnie HYN, Balasubramanian R (2011). Biosorption characteristics of crab shell particles for the removal of manganese(II) and zinc(II) fromaqueous solutions. Desalination.

[59] Ucer A, Uyanik A, Aygun SF (2006). Adsorption of Cu (II), Cd (II), Zn (II), Mn (II) and Fe (III) ions by tannic acid immobilized activated carbon. Sep. Purif. Technol..

[60] Taffarel SR, Rubio J (2010). Removal of Mn2+ from aqueous solution by manganese oxide coated zeolite. Miner. Eng..

