# Supplementary material for: Selective removal of some heavy metals from Lanthanide solution by graphene oxide functionalized with sodium citrate
Source: Sci Rep. 2022 Aug 12;12:13755. doi: 10.1038/s41598-022-17949-8 (PMC9374659; doi:10.1038/s41598-022-17949-8)
Supplement: Supplementary file 1 — Supplementary Information. [file 41598_2022_17949_MOESM1_ESM.docx]

**Supplementary Materials**

**Selective removal of some heavy metals from Lanthanide solution by graphene oxide functionalized with sodium citrate**

E.M. Abu Elgoud^a^, A.I. Abd-Elhamid^b^, Sh.Sh. Emam^a^, H.F. Aly^a^

^a^Hot Laboratories Center, Egyptian Atomic Energy Authority, 13759, Egypt

^b^ Composite and nanostructured materials Department, Advanced Technology and New Materials Research Institute, City of Scientific Research and Technological Applications (SRTA-City), New Borg Al-Arab, Alexandria 21934, Egypt

**2. Experimental**

**2.3.1. Kinetic study**

The adsorption of metal ions over the GO-C show rapid equilibrium. Therefore, the Pseudo-first-order kinetic model was not involved. The pseudo-second-order equation investigated in a term:

$\frac{\text{t}}{q_{t}}=\frac{1}{K_{2 }q_{e}^{2}}+\frac{t}{q_{e}}$ (2)

Where K_2_ (g mg^-1^min^-1^) is the rate constant of pseudo-second-order reaction, and q_e_ (mg/g) is the number of sorbet species at equilibrium time (q_t_) in (mg/g) is the content of sorbet species at a time (t).

**2.3.2. Adsorption isotherm**

**2.3.2.1. Freundlich isotherm model**

Freundlich linear form can be expressed as follows, **Eq. 3** ^1^:

Where *K_f_* is a constant that indicates the relative sorption capacity of the sorbet ions (mg/g) and 1/n is a constant indicating the intensity of the sorption process. The linearized isotherm plots are shown in Fig. 5b. The correlation coefficient R^2^ and numerical values of Freundlich constants were calculated and listed in Table 3. The low value of the correlation coefficient (*R^2^*) illustrates that the sorption of the studied ions does not obey Freundlich isotherm on the range of the studied sorption concentration.

**2.3.2.2. Langmuir isotherm model**

This model presents a monolayer form of adsorbate that is sorbed on energetically identical binding sites. The Langmuir equation is presented linearly in **Eq. 4** ^1^ as follows:

Where *Q^o^* is the monolayer sorption capacity (mg/g), and *b* is the Langmuir equilibrium constant belonging to sorption energy (L/mg). The plotting of (*C_e_/q_e_*) versus *C_e_* gives straight lines for all metal ions, as represented in Fig.5c. The R^2^ and calculated Langmuir relation constants *Q^o^*, and b were tabulated in Table 3. The value of R^2^ for all the studied metal ions was more than 0.998, suggesting that the Langmuir model can describe the adsorption isotherm model of the (Fe^3+^, Ni^2+^, and Mn^2+^) ions on the GO-Cit. Additionally, the maximum adsorption capacities (mg g^-1^) were found at 531.91 (Fe^3+^), 171.23 (Ni^2+^), and 223.22 (Mn^2+^). Moreover, the sorption Langmuir energy (b) values for the five studied metals were greater than zero, explaining that Langmuir is an appropriate model ^2^.

One of the important characteristics of the Langmuir equation is an equilibrium parameter, *R_L_*, a dimensionless constant, and can be described by the following relation **Eq. 5** ^1^:

The values of *R_L_*, indicates the type of isotherm to be unfavorable (*R_L_*> 1), irreversible (*R_L_* = 0), favorable (0 <*R_L_*< 1) or linear (*R_L_* = 1). The three studied metals own *R_L_* of more than zero and less than a unit, indicating a favourable adsorption reaction.

**2.3.2.3. Dubinin–Radushkviech isotherm model (D–R isotherm)**

The D–R isotherm model describes the sorption process on homogeneous and heterogeneous binding surface sites. Moreover, it is applied to differentiate between the physical and chemical sorption ^3^. The D–R isotherm was verified in the form **Eq. 6**, ^1^:

Where *q*_m_ (mmol/g) is the maximum amount of sorbate that can be adsorbed onto the unit weight of the adsorbent, *β* is the constant related to the sorption energy (mol^2^/kJ^2^), and ε is a Polanyi potential. The theory of Polanyi assumes that the existence of a constant volume of sorption space locate near the surface of the sorbent and the sorption potential takes place at these spaces ^4^. The value of *ε* can be calculated from **Eq. 7:**

Where *R* is the gas constant equal to 0.008314 (kJ/mol K), *T* is the solute temperature (K), and *C_e_* is the equilibrium concentration of ions (mmol /L). Plotting of ln *q_e_* against *ε^2^*, Fig.5d, from the slope and intercept, *β* and *q_m_* can be evaluated, respectively, as listed in Table 3. The mean free energy, *E*, is the free energy change required for one mole of ions to travel from the bulk of the solution to the sorbent surface ^5^. It donated an explanation of the sorption mechanism; if the *E* value lies in the range 8.0-16.0 kJ mol^−1^, the sorption operation could be described as ion-exchange or chemisorption, based on the alter in the bulk solution pH value at equilibrium. While if *E*< 8.0 kJ mol^−1^, the sorption process has physical nature ^5^. The mean free energy *E* (kJ/mol) of the adsorption process can be estimated by **Eq. 8** ^1^:

The values of q_m_, β, E, and the correlation factor (*R*^2^) parameters for Fe, Ni, and Mn ions are summarized in Table 3. The values of the mean free energy, *E*, kJ/mol, of sorption in all cases are in the range 10.31 (Fe), 12.23 (Ni), and 13.2 (Mn), which are within the ranges of chemical sorption reaction, whereas, no variation in pH of the solution at equilibrium was detected.

**2.3.2.4. Flory–Huggins isotherm model**

Flory–Huggins isotherm model ^6^ was applied to derive the ratio of surface coverage characteristics of adsorbate onto adsorbent. Moreover, it can explore the availability and spontaneous nature of the adsorption process. The linearized form of the Flory–Huggins isotherm model is presented by the following relation **Eq. 9:**

In this respect, θ is the degree of surface coverage, where θ = 1-(C_e_/C_o_). K_FH_ and n_FH_ indicate its equilibrium constant and model exponent, respectively. Plotting of log (θ/C_o_) against log (1-θ), Fig.5e, the n_FH_ and K_FH_ could be determined from the slope and intercept, respectively, and summarized in Table 3.

Moreover, K_FH_, used for the determination of spontaneity free Gibbs energy (Table 3), using the following **Eq. 10** ^7^:

ΔG◦ = −RT ln (K_FH_) (10)

**2.3.2.5. Temkin isotherm model**

The Temkin isotherm model assumes that surface coverage due to adsorbent–adsorbate interactions will be led to linearly decreases in the adsorption energy. The linear form of the Temkin isotherm model is given by the  **Eq. 11** ^7^:

The plot of q_e_ against ln C_e_ for Fe^3+^, Ni ^2+^ and Mn ^2+^ sorption is represented in Fig.5f. From the slope and intercept of the plot, the Temkin isotherm equilibrium binding constant (L/g) for monolayer adsorption capacity K_t_ (L/g) and the Temkin isotherm constant (b_t_) were evaluated and listed in Table 3.

**2.3.3. Thermodynamic Isotherm**

The standard thermodynamic parameters of the sorption process are represented through Gibbs free energy, ΔG°, Enthalpy, ΔH°, and Entropy, ΔS°. These represent the main thermodynamic function of assessing sorption reaction. Vant Hoff equation was used to evaluate Gibbs free energy as presented, ^1^:

Where *K_d_* is the sorption equilibrium constant of the sorption process, *T* is the absolute temperature, K, and *R* is the universal gas constant, 8.314 J/mol/K. The enthalpy change, *ΔH°* was obtained from the following **Eq. 13** ^1^:

**3. Results and Discussion**

**3.3.1. Error functions analysis**

There are different error functions required to evaluate isotherm models ^8,9^. If information from the models is closed to the experimental data, the error function value will be a small number. If they are different, the error function value will be a large number, and the small the error function value, the better the curve fitting. In general, plus values of errors, the function is low, which means there is an agreement between the experimental and calculated data and a more favourable model. From Table 4 according to the error function, as well as R^2^, it is clear that Langmuir is the best model to describe the adsorption data.

**Table S1:** The isotherm constants obtained by different error function and their R^2^ value.

| **Error function** | **Langmuir** | **Freundlich** | **D-R** | **Temkin** |
| --- | --- | --- | --- | --- |
| **Adj. R-Square (R^2^)** | 0.999 | 0.953 | 0.968 | 0.984 |
| **Root mean square deviation (RMSD)** | 4.9 | 297.8 | 523.4 | 67.3 |
| **Root mean square error (RMSE)** | 8.5 | 515.9 | 906.5 | 116.5 |
| **Nonlinear chi-square test (χ2)** | 0.08 | 285.3 | 880.92 | 14.6 |
| **Sum of the squares of the errors (ERRSQ)** | 72.1 | 266150.5 | 821791.4 | 13579.24 |
| **Average relative error (ARE)** | 1.9 | 79.2 | 118.4 | 15.6 |
| **Sum of absolute error (EABS)** | 14.0 | 767.6 | 1240.4 | 162.5 |
| **Hybrid fractional error function (HYBRID)** | 5.7 | 237.7 | -355.179 | 46.7 |
| **Marquardt’s percent standard deviation (MPSD)** | 3.7 | 137.7 | 214.1 | 27.7 |
| **Average percentage error (APE)** | 2.9 | 118.8 | 177.6 | 23.4 |

**
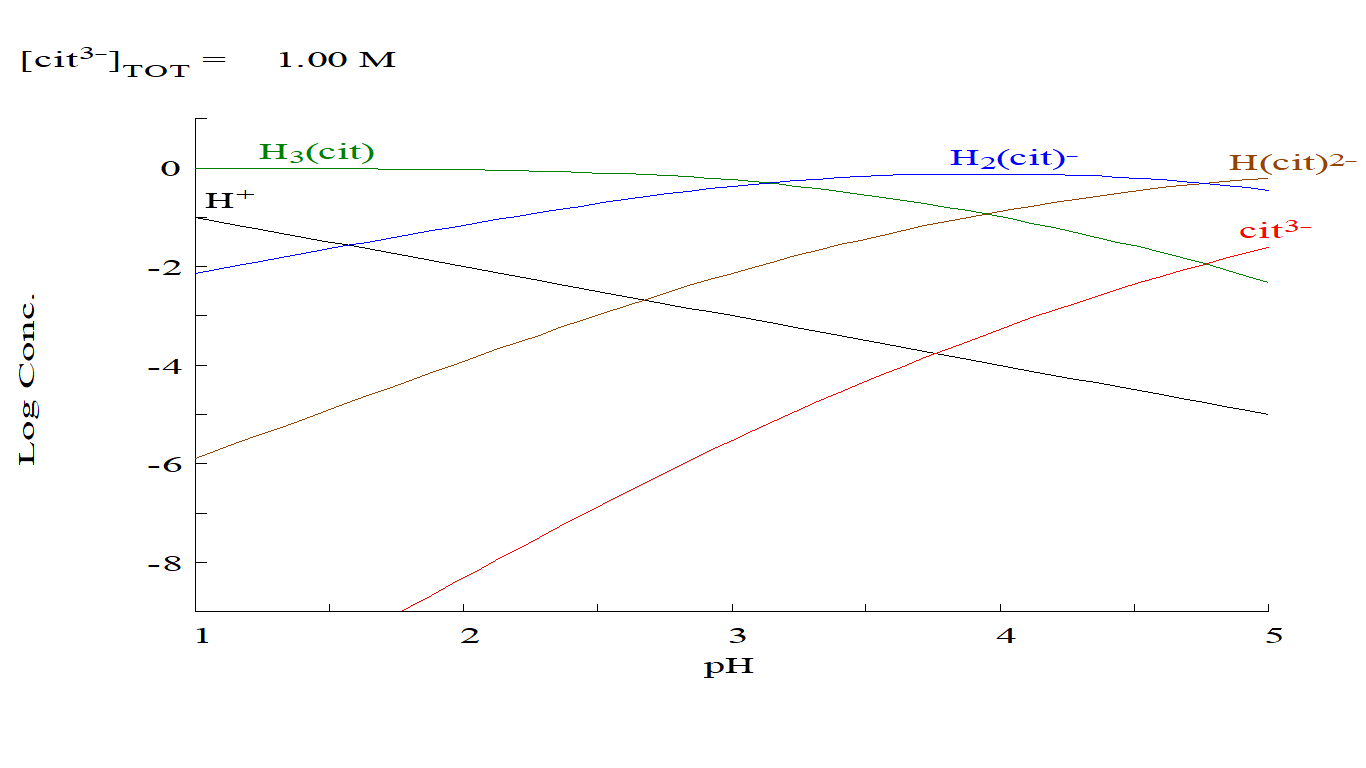
**

**Figure S1.** Equilibrium diagram of citric acid in pH range (1.0 -5.0) [**www.kemi.kth.se/medusa**](http://www.kemi.kth.se/medusa)**.**

**Reference**

1. E.M. Abu Elgoud, Z.H. Ismail, M.I. Ahmad, Y.A. El–Nadi, S.M. Abdelwahab, H.F. Aly,Sorption of lanthanum(III) and neodymium(III) from concentrated phosphoric acid bystrongly acidic cation exchange resin (SQS-6) Russ. J. Appl. Chem. 92 (2019) 1581−1592.
2. Metwally, S.S., Ayoub, R.R., Aly, H.F. (2013). Amidoximation of cyano group for chelating ion exchange of some heavy metal ions from waste water. Sep. Sci. Technol. 48(12), 1830–1840.
3. Metwally, S.S., Rizk, H.E. (2014). Preparation and characterization of nano-Sized iron–titanium mixed oxide for removal of some lanthanides from aqueous solution. Sep. Sci. Technol. 49(15), 2426–2436.
4. Keereeta, Y., Thongtem, T., Thongtem, S., (2015). Synthesis of lanthanum tungstate interconnecting nanoparticles by high voltage electrospinning. Appl. Surf. Sci. 351, 1075–1080.
5. El-Gammal, B., Metwally, S.S., Aly, H.F., Abo-El-Enein S.A. (2012). Verification of double-shell model for sorption of cesium, cobalt, and europium ions on poly-acrylonitrile-based Ce(IV) phosphate from aqueous solution. Desalin. Water Treat. 46(1–3), 124–138.
6. M. Horsfall, A.I. Spiff, Equilibrium sorption study of Al3+, Co2+ andAg2+ in aqueous solutions by fluted pumpkin (Telfairia occidentalis HOOK) waste biomass, Acta Chim. Slov. 52 (2005) 174–181.
7. K.Y. Foo, B.H. Hameed “Insights into the modeling of adsorption isotherm systems” Chemical Engineering Journal 156 (2010) 2–10.
8. M. Hamzaoui, B. Bestani, N. Benderdouche “The use of linear and nonlinear methods for adsorption isotherm optimization of basic green 4-dye onto sawdust-based activated carbon” J. Mater. Environ. Sci., 2018, Volume 9, Issue 4, Page 1110-1118.
9. Hyndman, R.J., Koehler, A.B. (2006). Another look at measures of forecast accuracy. Int. J. Forecasting. 22(4), 679–688.
